# Bi-directional causal effect between vitamin B12 and non-alcoholic fatty liver disease: Inferring from large population data

**DOI:** 10.3389/fnut.2023.1015046

**Published:** 2023-03-06

**Authors:** Liwan Fu, Yuquan Wang, Yue-Qing Hu

**Affiliations:** ^1^Center for Non-Communicable Disease Management, Beijing Children’s Hospital, National Center for Children’s Health, Capital Medical University, Beijing, China; ^2^State Key Laboratory of Genetic Engineering, School of Life Sciences, Human Phenome Institute, Institute of Biostatistics, Fudan University, Shanghai, China; ^3^Shanghai Center for Mathematical Sciences, Fudan University, Shanghai, China

**Keywords:** non-alcoholic liver disease, vitamin B12 concentrations, liver enzymes, Mendelian randomization, genome-wide association studies

## Abstract

**Objectives:**

Many observational studies evaluate the association between vitamin B12 and non-alcoholic fatty liver disease (NAFLD). However, the causality of this association remains uncertain, especially in European populations. We conducted a bidirectional Mendelian randomization study to explore the association between vitamin B12 and NAFLD.

**Methods:**

Two-sample Mendelian randomization study was conducted. Summary statistics for vitamin B12 were acquired from a genome-wide association studies (GWAS) meta-analysis including 45,576 subjects. Summary-level data for NAFLD was obtained from a GWAS meta-analysis of 8,434 cases and 770,180 non-cases and another GWAS meta-analysis of 1,483 cases and 17,781 non-cases. Summary-level data for 4 enzymes including alkaline phosphatase (ALP), alanine aminotransferase (ALT), aspartate aminotransferase (AST), and gamma glutamyltransferase (GGT), was available from the UK Biobank. Inverse variance weighting (as main analysis), weighted median estimate, robust adjusted profile score, MR-Egger, and MR-PRESSO (sensitivity analyses) were performed to calculate causal estimates.

**Results:**

Genetically predicted higher vitamin B12 concentrations were consistently associated with an increased NAFLD in two sources. The combined odds ratio (OR) of NAFLD was 1.30 (95% confidence interval (CI), 1.13 to 1.48; *p* < 0.001) per SD-increase in vitamin B12 concentrations. Genetic liability to NAFLD was also positively associated with vitamin B12 concentrations (Beta 0.08, 95%CI, 0.01 to 0.16; *p* = 0.034). Sensitivity analyses also revealed consistent results. Genetically predicted vitamin B12 concentrations showed no significant association with liver enzymes.

**Conclusion:**

The present study indicates that increased serum vitamin B12 concentrations may play a role in NAFLD risk. NAFLD also has a causal impact on elevated vitamin B12 concentrations in the circulation. Notably, vitamin B12 concentrations imply the levels of vitamin B12 in the circulation, and higher intake of vitamin B12 may not directly lead to higher levels of serum vitamin B12, instead the higher levels of vitamin B12 in the circulation may be caused by the dysregulation of the metabolism of this vitamin in this study. There exist bidirectional causal effects between serum vitamin B12 concentrations and risk of NAFLD in European individuals.

## 1. Introduction

Non-alcoholic fatty liver disease (NAFLD), as the leading liver disease worldwide, influences approximately 25% of the world population (1). Due to the sedentary lifestyle, western diet, and obesity epidemic, the prevalence of NAFLD keeps increasing (2). NAFLD, mostly encompassing non-alcoholic steatohepatitis (NASH), isolated hepatic steatosis, and cirrhosis (3), is considered as a feature of metabolic syndrome in the liver (4). As NAFLD is always symptomless and difficult to be observed, it is hard to predict the progression of NAFLD (5). Thus, identifying potential biomarker is needed to predict the emergence and development of NAFLD.

Vitamin B12, mainly presenting two forms in humans: 5′-deoxyadenosylcobalamine and methyl cobalamin, was reported to be correlated with hepatitis and cirrhosis (6). In addition, vitamin B12 was served as a cofactor for methyl malonyl CoA mutase, which managed the rate of long-chain fatty Acyl-CoA enter into mitochondria and influences lipid metabolic pathways (7). The liver was served as a storage site for vitamin B12. An increased serum content of vitamin B12 found in acute and chronic liver diseases has been attributed to the release of the vitamin from the liver owing to hepatic necrosis and/or to an increased capacity of the serum to bind vitamin B12 as a result of abnormalities in the serum proteins. Moreover, several studies, in animal models, investigated the impact of vitamin B12 change in lipid metabolism (8). The liver is an important organ for lipid metabolism. Thus, it is reasonable to suppose that serum vitamin B12 concentrations may change in the occurrence of liver injury. Importantly, vitamin B12 is a water soluble nutrient which plays an important role in human health. Because of its water solubility, its intake is generally not excessively restricted. Dietary intake of vitamin B12 is indispensable to the maintenance of human health and deficiencies can result in severe health consequences. Notably, it does not necessarily imply that higher levels of serum vitamin B12 is due to a higher intake, instead the dysregulation of the metabolism of this vitamin may be the driver of higher levels of vitamin B12 in the circulation. Vitamin B12 is cofactor for enzymes in one-carbon metabolism, which plays a central role in the generation of methyl donors in the form of S-adenosylmethionine (SAM), the sole methyl donor used by DNA, RNA, histone, and protein methyltransferases (9). Therefore, the intake of vitamin B12 should be given great attention, especially its impact on NAFLD. Moreover, some study designs, including cross-sectional and case–control studies, have evaluated the association between vitamin B12 and NAFLD (10–22). Several studies implicated positive association between vitamin B12 and NAFLD (12, 16, 20, 21), whereas others indicated inverse association (10, 13, 19) or no association (11, 14, 15, 17, 18, 22). Notably, a latest systematic review and meta-analysis comprising 361 NAFLD subjects and 510 controls demonstrated no association of vitamin B12 concentrations with risk of NAFLD (23). Nevertheless, a variety of limitations, including a great heterogeneity in different studies, selection bias, and confounding factors, were not fully considered and described in its interpretation (23). Importantly, potential reverse causality and residual confounding were unable to be explained by these observational studies, so aforementioned controversial findings deriving from observational studies could not provide the causal inference in the association between vitamin B12 concentrations and NAFLD.

Leveraging genetic variants as instrumental variables for an exposure (e.g., vitamin B12), Mendelian randomization (MR) was able to strengthen causal inference of an exposure-outcome association *via* diminishing reverse causality and residual confounding (24). Additionally, the sample size of the population, and the relationship between vitamin B12 concentrations and NAFLD differing between ethnicities need to be further considered (20, 22, 23). Therefore, we performed a bidirectional 2-sample MR study on the basis of large populations to investigate this relationship in the European population. Concurrently, we also carried out a 2-sample MR study to estimate the association of vitamin B12 concentrations with NAFLD-related liver enzymes.

## 2. Materials and methods

### 2.1. Study design

**Figure 1:**
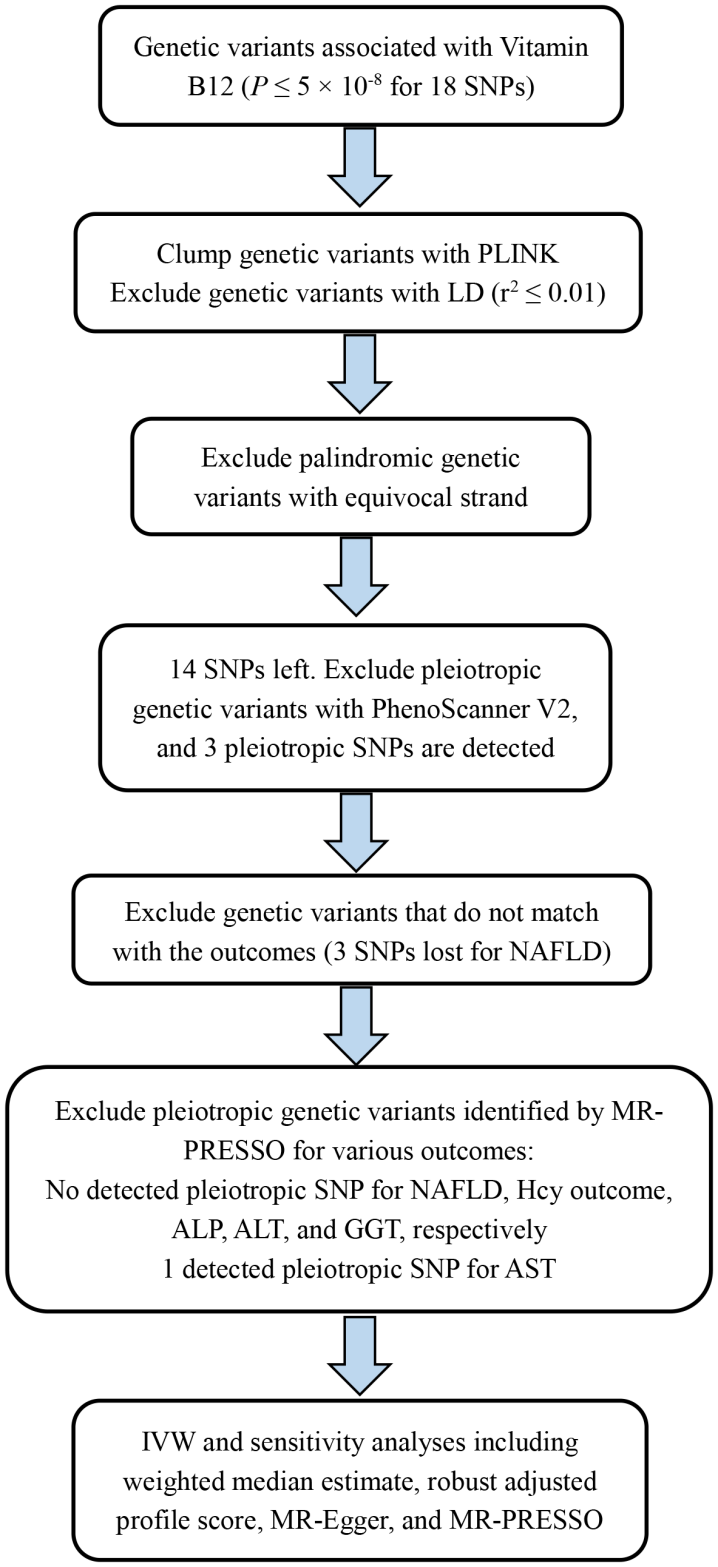
Flow chart of Vitamin B12 as exposure in this Mendelian randomization. ALP, alkaline phosphatase; ALT, alanine aminotransferase; AST, aspartate aminotransferase; GGT, gamma glutamyltransferase; Vitamin B12, serum Vitamin B12; IVW, inverse variance weighted; MR-PRESSO, Mendelian randomization pleiotropy residual sum and outlier; NAFLD, non-alcoholic fatty liver disease.

We performed this MR study based on summary statistics of genome-wide association analyses on serum vitamin B12 levels, NAFLD, and liver enzymes from large-scale genome-wide association studies (GWAS) (25–28), which included different studies and consortia encompassing the UK Biobank study, the Estonian Biobank, the FinnGen consortium, and so on (Supplementary Table S1). We first calculated genetic correlations of vitamin B12 concentrations with NAFLD and liver enzymes. Second, we conducted a forward MR analysis to evaluate the causal effect of genetic prediction of higher vitamin B12 concentrations on NAFLD risk and concentrations of liver enzymes. Considering the possibility that NAFLD might impact vitamin B12 concentrations as the liver is regarded as a storage site for vitamin B12 (6), we performed a reverse MR analysis to investigate the association between genetic liability to NAFLD and vitamin B12 concentrations. The detailed flow chart of vitamin B12 as exposure in this MR study is displayed in Figure 1. All the studies comprised in cited GWAS were approved by a relevant review board. No necessary for ethical permit in this MR analysis because of summary-level data.

### 2.2. Data sources of vitamin B12

We identified 18 single nucleotide polymorphism (SNPs) associated with serum vitamin B12 concentrations from a GWASs meta-analysis including 45,576 individuals of European descent (25) at the genome-wide significant level (*p* value less than 5E-08). Based on 1,000 Genomes European reference panel, we used the PLINK clumping technique to estimate linkage disequilibrium among these 18 SNPs. Consequently, 14 independent SNPs without linkage disequilibrium (*r*^2^ < 0.01) were chosen as alternative instruments for vitamin B12. Covariates, such as sex, age, and principal components, were adjusted in the corresponding association estimates (Supplementary Table S1). These instruments explain about 6.0% of phenotypic variance for vitamin B12 and have been employed in previous MR studies (29, 30). We used the internet resource (PhenoScanner V2) (31) to test whether these 14 instruments are correlated with other phenotypes, and noticed that 3 SNPs (rs1131603, rs2336573, and rs602662) were associated with other phenotypes (Supplementary Table S2, the search was conducted in April 2022). These 3 SNPs probably exerted pleiotropic effects and were removed, plus 3 other SNPs that do not match with NAFLD (Supplementary Table S3), and a total of 8 independent SNPs were selected as instruments for vitamin B12 eventually. As the exposure, vitamin B12 concentrations were transformed to one standard deviation (SD) unit (equivalent to standardization). For testing the strength of genetic instruments, we calculated *F*-statistics for vitamin B12 (*F* = 242.4), which was greater than 10, suggesting the support for the strength of genetic instruments. For the reverse MR study, summary-level data for vitamin B12 concentrations was obtained from a study from Vanderbilt University Medical Center (27) because of scanty SNPs associated with NAFLD were selected from this GWAS meta-analysis (25) when NAFLD was acted as an exposure.

### 2.3. Data sources of non-alcoholic fatty liver disease

Summary data for the associations of vitamin B12 associated SNPs with NAFLD was obtained from two large meta-analyses of GWAS. One GWAS was from Ghodsian et al. (28), which encompassed 8,434 cases and 770,180 controls of European descent. This GWAS meta-analysis included four cohorts: the UK Biobank, FinnGen, the Estonian Biobank and the Electronic Medical Records and Genomics (eMERGE) (32). The eMERGE defined NAFLD cases by Electronic health record (EHR) codes (ICD9: 571.5, ICD9: 571.8, ICD9: 571.9, ICD10: K75.81, ICD10: K76.0 and ICD10: K76.9). NAFLD in the UK Biobank, FinnGen and the Estonian Biobank were defined by International Classification of Disease code K76.0. In these four cohorts, the associations were adjusted for age, gender, 10-main ancestry-based principal components and genotyping batch. The other GWAS from Anstee et al. (26) included 1,483 NAFLD cases and 17,781 controls of European descent. NAFLD cases in this GWAS were defined on the basis of abnormal biochemical estimates and/or an ultrasonographically tested bright liver, along with diagnostics of the metabolic syndrome; or observing abnormal biochemical tests and macroscopic appearances of a steatotic liver at the time of bariatric surgery (26). Specific stages of NAFLD and histology are available in GWAS from Anstee et al., and the first 5 principal components were adjusted for the associations in Anstee et al., GWAS.

For the reverse MR analysis, 6 SNPs associated with NAFLD at the genome-wide significant level (*p* < 5E-08) were selected as instruments from the Ghodsian et al., GWAS (28). As rs58542926 was strongly correlated with rs10401969, we removed s58542926 and 5 independent SNPs were left as instruments in the reverse MR analysis eventually (Supplementary Table S4). Additionally, we performed a sensitivity analysis of the reverse MR by utilizing SNPs as instruments from the Anstee et al., GWAS at the genome-wide significant level. As a result, 6 independent SNPs (*r*^2^ < 0.01) were chosen as instruments in the sensitivity analysis of the reverse MR (Supplementary Table S5).

### 2.4. Data sources of liver enzymes

We acquired four liver enzymes, including alkaline phosphatase (ALP), alanine aminotransferase (ALT), aspartate aminotransferase (AST), and gamma glutamyltransferase (GGT), which were possibly correlated with NAFLD (28). Summary data for the associations between vitamin B12 associated SNPs and these liver enzymes was obtained from the outcomes of the second wave in the UK Biobank through the Neale lab (Supplementary Table S6).

### 2.5. Statistical analysis

Overlapping samples between two datasets could result in bias for the estimated causal effects. Thus, we utilized linkage disequilibrium score regression (LDSC) to estimate sample overlap with LD hub^1^ (33). Inverse variance weighting (IVW) with multiplicative random was used as the main analysis in this study (34). Then, we combined estimates from Ghodsian et al., GWAS and Anstee et al., GWAS *via* the fixed-effects meta-analysis method. Four MR approaches encompassing weighted median estimate (35), robust adjusted profile score (36), MR-Egger (37), and MR-PRESSO (38), were conducted as sensitivity methods for evaluating the consistency of results or correcting for pleiotropy. The weighted median estimate could provide an unbiased test when 50% SNPs are invalid instruments (35). Robust adjusted profile score correctly considered the measurement error in the selected instruments to acquire precise estimates with smaller bias (36). MR-Egger offered an assessment for horizontal pleiotropy *via* the *p* value of its intercept, and a test was obtained after the pleiotropic effects were adjusted. However, wider confidence intervals (CIs) were got due to a loss of statistical power (37). MR-PRESSO is another method evaluating biases caused by pleiotropy (through the global test). It gives a corrected estimate by removing the outliers, and also provides a distortion test, which estimates whether the results with or without outliers were different (38). We performed Cochran’s Q statistic to test the magnitude of heterogeneity (39) for the included SNPs. All analyses were conducted by R Version 4.1.0 utilizing R packages (“TwoSampleMR”) (40) and (“MRPRESSO”) (38).

## 3. Results

### 3.1. Evaluation for sample overlap

LDSC was conducted to evaluate sample overlap between exposure GWAS and outcome GWAS. Results showed approximately zero intercept of genetic covariance in pairs of exposure-outcome GWAS (*p* > 0.1 through z-test in all pairs, data not displayed), indicating no sample overlap in pairs of two GWAS datasets in this study.

### 3.2. Forward Mendelian randomization analysis

**Figure 2:**
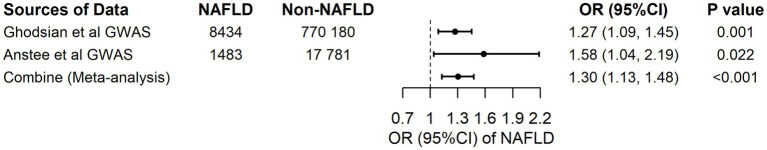
Association between genetically predicted Vitamin B12 and NAFLD. Vitamin B12, serum Vitamin B12; GWAS, genome-wide association studies; OR, odds ratio; NAFLD, non-alcoholic fatty liver disease.

Firstly, we used Ghodsian et al., GWAS data to perform the forward MR analysis. As a result, genetic prediction of higher vitamin B12 concentrations was associated with an elevated risk of NAFLD (odds ratio (OR) per 1-SD increase, 1.27; 95% confidence interval (CI), 1.09 to 1.45; *p* = 0.001) by the IVW-multiplicative random effect model. Then, the significance of the association was replicated in Anstee et al., GWAS (OR = 1.58; 95%CI, 1.04 to 2.19; *p* = 0.022) (Figure 2). Secondly, we combined tests from these two GWAS data sources with the fixed-effect model in the meta-analysis, and the combined OR of NAFLD was 1.30 (95%CI, 1.13 to 1.48; *p* < 0.001) for a 1-SD elevation in genetic prediction of vitamin B12 concentrations (Figure 2). All the sensitivity analyses also showed significant results (Table 1). We found no evidence of heterogeneity for the included SNPs (Both Cochrane’s Q < 5 from Ghodsian et al., GWAS and Anstee et al., GWAS data source), and MR-Egger showed no horizontal pleiotropy (Table 1). Additionally, no outlier was identified during the MR-PRESSO analysis.

**Table 1:** Association of genetic prediction of serum vitamin B12 levels with non-alcoholic fatty liver disease in sensitivity analyses.

Outcome source	Method	OR	95%CI	*P*
Ghodsian et al., GWAS (8,434 cases, 770,180 controls)	IVW-multiplicative random effects	1.27	1.09–1.45	0.001
	Weighted median estimate	1.30	1.07–1.54	0.004
	Robust adjusted profile score	1.27	1.08–1.46	0.001
	MR-PRESSO	1.27	1.16–1.38	0.001
	Cochrane’s Q = 2.58 (*p* = 0.92); MR-Egger intercept = −0.006 (*p* = 0.576)			
Anstee et al., GWAS (1,483 cases, 17,781 controls)	IVW-multiplicative random effects	1.58	1.04–2.19	0.022
	Weighted median estimate	1.79	1.11–2.60	0.016
	Robust adjusted profile score	1.58	1.06–2.19	0.023
	MR-PRESSO	1.57	1.16–2.04	0.022
	Cochrane’s Q = 4.28 (*p* = 0.746); MR-Egger intercept = −0.012 (*p* = 0.857)			

CI, confidence interval; GWAS, genome-wide association study; IVW, inverse variance weighted; MR, Mendelian randomization; MR-PRESSO, Mendelian randomization pleiotropy residual sum and outlier; OR, odds ratio.

Based on the UK Biobank data, genetic prediction of vitamin B12 concentrations showed no association with liver enzymes in the main analysis (IVW-multiplicative random effects). Other MR approaches revealed similar estimates in the sensitivity analyses (Supplementary Figure S1), except for negative association with GGT through robust adjusted profile score method (Beta, −0.69; 95%CI, −1.26 to-0.13; *p* = 0.016). Moderate heterogeneity was observed in the main analysis of AST, while no pleiotropy was detected in the MR-Egger analysis.

### 3.3. Reverse Mendelian randomization analysis

**Figure 3:**
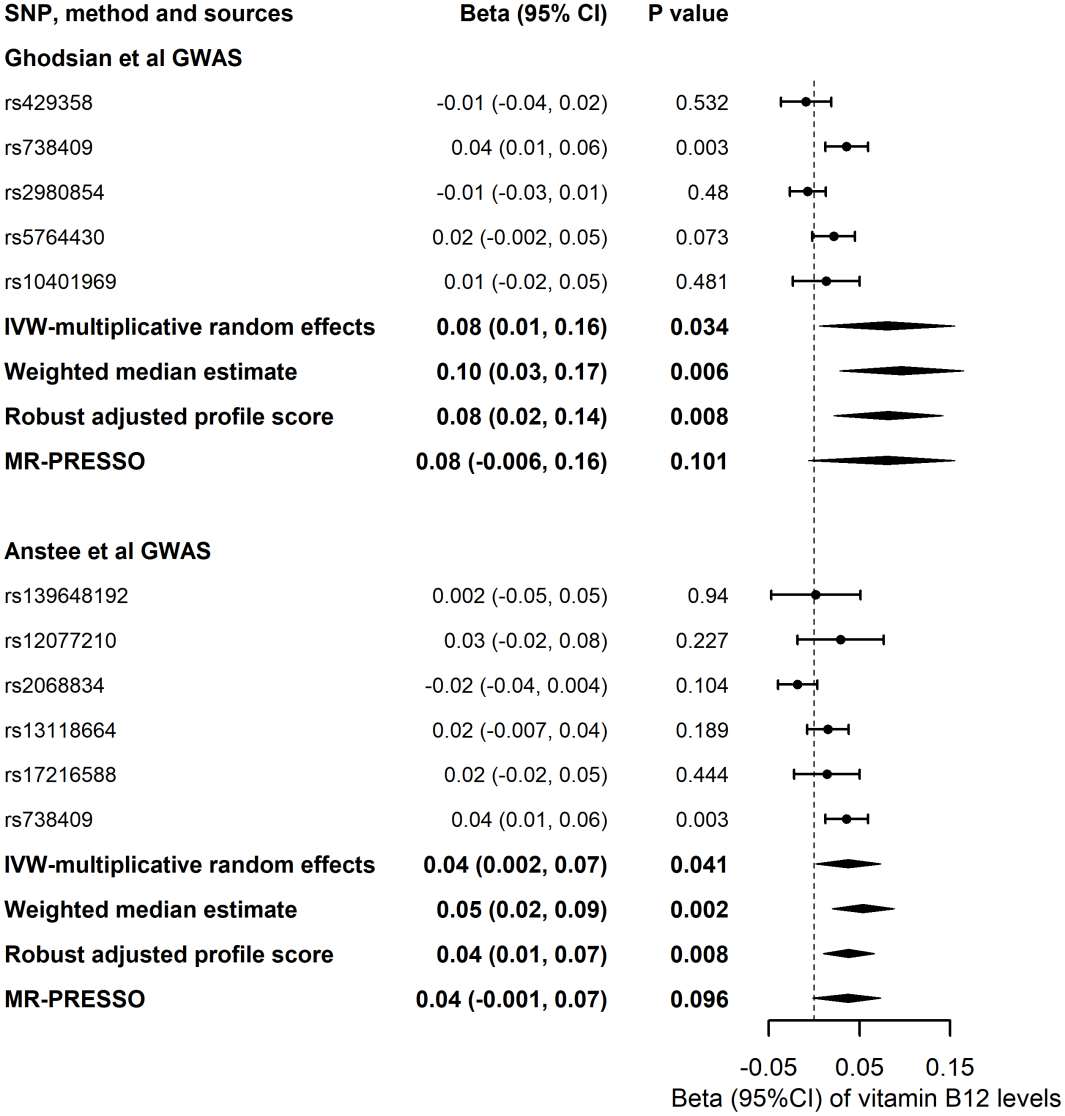
Association of genetic liability to NAFLD with Vitamin B12. Vitamin B12, serum Vitamin B12; IVW, inverse variance weighted; MR-PRESSO, Mendelian randomization pleiotropy residual sum and outlier.

In the main analysis, liability to NAFLD revealed positive association with vitamin B12, and the effect size of vitamin B12 concentrations in 1-SD change was 0.08 (95%CI, 0.01 to 0.16; *p* = 0.034) for 1-unit increase in the log-transformed OR of NAFLD (Figure 3). Other MR approaches persistently showed the significant association (Figure 3). No horizontal pleiotropy was detected in the MR-Egger analysis (*P* for the intercept was 0.28), and no outlier was identified during the MR-PRESSO analysis. We further employed 6 SNPs detected from Anstee et al., GWAS data for the sensitivity analysis and observed that liability to NAFLD also had positive association with vitamin B12 concentrations (Beta, 0.04; 95%CI, 0.002 to 0.07; *p* = 0.041) by the main analysis and other MR approaches (Figure 3).

## 4. Discussion

The present study elucidated positive genetic associations between serum vitamin B12 and NAFLD risk. Forward MR analysis showed that genetic prediction of increased serum vitamin B12 concentrations were robustly associated with an elevated risk of NAFLD. Moreover, the reverse MR analysis was able to give support for an effect of NAFLD on serum vitamin B12 concentrations. Our findings indicated that higher serum vitamin B12 was a strong casual factor of increased risk of NAFLD, which in turn would further increase serum vitamin B12.

Previous studies on the relationship between vitamin B12 concentrations and NAFLD were controversial. A meta-analysis encompassing 8 cross-sectional and case–control studies published recently found that NAFLD cases had same vitamin B12 concentrations compared with those without NAFLD (23). No association was implicated in earlier studies (11, 14–18). In contrast, a study involved in 614 Brazilian cases reported that higher serum concentrations of vitamin B12 were positively associated with the severity of fibrosis and steatosis (20). Vitamin B12 acts as a coenzyme for a crucial methyl transfer reaction, and the recommended dietary allowance for adult reaches 2.4 μg of vitamin B12 per day (41). Dietary intakes of vitamin B12 were also found increase in patients with NAFLD compared with controls in 101 Canadians (42) and 120 adult Jordanians (43), which were consistent with our main result that elevated vitamin B12 was causally associated with the increased risk of NAFLD. Nevertheless, both dietary intakes of vitamin B12 and serum concentrations of vitamin B12 were unable to observe significant associations between NAFLD patients and controls in 317 Iranians (44) and 54 participants in Greece (15), respectively. Otherwise, serum concentrations of vitamin B12 were found in a lower level in NAFLD cases compared with those of the controls in 75 Turks (13), and decreased vitamin B12 concentrations were significantly associated with an increased fibrosis grade, as well as non-alcoholic steatohepatitis in 83 cases in Israel (45). However, previous publications on vitamin B12 and NAFLD usually contained a small number of subjects and did not consider covariates in univariate analysis. Previously, we have established genetic statistics to detect genetic variation in complex diseases and used MR approaches to evaluate the causal relationships between complex diseases, including the casual association of Hcy with NAFLD (46–54). Recently, a national population-based survey from NHANES showed positive associations of vitamin B12 with liver steatosis and fibrosis linearly (21), which are consistent with our findings to some degree. Additionally, different races, and distinct diagnostic criteria may be reasons for conflicting results in different studies. In contrast, our study on the basis of several large populations of European descent displayed a significant positive association of genetically predicted vitamin B12 concentrations with NAFLD risk. Inadequate power or unobserved confounding effects would contribute to the lack of association. Compared to aforementioned cross-sectional and case–control studies, we performed several large populations encompassing over approximately 10,000 NAFLD patients and over 780,000 controls, conducted state-of-art approaches of causal analysis (various MR methods) to minish reverse causality and unobserved confounding impact, and eventually, offered sufficient power to evaluate the causal effect of vitamin B12 concentrations on NAFLD risk.

Interestingly, our study was able to establish a significant impact of genetic liability to NAFLD on increased serum concentrations of vitamin B12. Similarly, the association of genetic liability to NAFLD with serum vitamin B12 concentrations was consistent in our sensitivity analyses. Therefore, bi-directional causal effects were observed, and instances of true bi-directional pathway might exist, and in other words, a proposed positive effect of vitamin B12 on NAFLD risk, whereas NAFLD also exerts a positive effect on vitamin B12, possibly as part of a positive feedback loop. For patients at high risk of NAFLD, this study might indicate that the intake of vitamin B12 may be restricted because it might elevate the serum vitamin B12 concentrations, and then increase the risk of NAFLD. However, the dysregulation in the pathways that regulate B12 metabolism may influence the serum level of vitamin B12, and the dysregulation of the metabolism of this vitamin may be the driver of higher levels of vitamin B12 in the circulation. Therefore, it does not necessarily imply that higher levels of serum vitamin B12 is due to a higher intake. A recent randomized controlled trial indicated that vitamin B12 supplementation significantly decreased serum levels of homocysteine compared to placebo, while no significant difference was revealed in between-group comparisons for fasting blood glucose, malondialdehyde, and liver steatosis (55). Because this study was unable to support our findings, further studies with different doses will reveal additional evidence. In addition, new trials with dose-responsive effect of serum vitamin B12 on NAFLD still need to be explored for this finding.

This MR analysis showed no association between genetic prediction of vitamin B12 concentrations and liver enzymes, which indicated that vitamin B12 was possibly unable to affect NAFLD risk through the pathways involved in liver enzymes. Extensive research has manifested the importance of vitamin B12 status in adjusting one-carbon metabolism (56, 57). As is known to all, the transsulfuration pathway is a part of one-carbon metabolism, and it acts as an important role in many progress of chronic diseases and has been correlated with inflammation, oxidative stress, ER stress, insulin resistance, portal hypertension, and steatosis (57). The relationship between the transsulfuration pathway and oxidative stress has been believed to be regulated through the adjustment of the antioxidant glutathione production. Experimental and animal studies demonstrated that the downstream products of vitamin B12 metabolism are increased and oxidative stress, steatosis, and fibrosis develop in the liver in cystathionine β-synthase-deficient mice (57). Furthermore, the lack of cystathionine β-synthase seemingly upregulates the expression of genes with regard to ER stress, hepatic lipid homeostasis, as well as genes linked to hepatic steatosis (57), whereas knockout of cystathionine γ-lyase results in decreased hepatic lipolysis (58). The aforementioned evidence may, to some extent, explain the possible mechanism by which vitamin B12 causes the risk of NAFLD.

Some strengths appear in the present study. The mentionable merit is MR design, which enhanced causal inference through decreasing reverse causation and residual confounding. Employing two large population-based GWAS meta for the association between genetic prediction of vitamin B12 concentrations and NAFLD, significantly improved the statistical power and solidified our results. Additionally, sensitivity analyses showed robust association and no unbalanced pleiotropy. Our findings were restricted to European populations and principal component analysis was adopted in the performed GWAS analyses. Thus, the population structure was possibly reduced. However, the population confinement blocked the generalizability of the present findings to other populations.

Limitations should also be mentioned when interpreting our results. As the lack of dose-responsive effect of vitamin B12 on NAFLD in this study, it is hard to apply the results of the present study to clinical practice without recommendation of a beneficial range. PhenoScanner V2 (31) was utilized to screen out the genetic instruments employed for vitamin B12 concentrations and associated with some phenotypes at the genome-wide significant level (Supplementary Table S2) for the horizontal pleiotropy concern. Even so, there probably still exist potential unobserved pleiotropic SNPs. It is worth noting that the definition of NAFLD patients was distinct between the Ghodsian et al., GWAS and Anstee et al., GWAS, which perhaps lead to heterogeneity in meta-analysis of associations in despite of a small degree. As previously mentioned, the associations of vitamin B12 concentrations with NAFLD may differ in populations of different ancestries. More studies focused on individuals of non-European descent are needed in the future. Last but not least, due to the lack of sex-stratified data in this study, whether the association of genetically predicted vitamin B12 concentrations with NAFLD risk differs between men and women needs further studies.

## 5. Conclusion

In conclusion, the present study unraveled positive relationship between vitamin B12 concentrations and NAFLD, and elucidated bidirectional causal effects between vitamin B12 concentrations and risk of NAFLD in European individuals. Significantly, vitamin B12 concentrations imply the levels of vitamin B12 in the circulation, and higher levels of serum vitamin B12 may not be directly caused by a higher intake, instead the dysregulation of the metabolism of this vitamin may contribute to higher levels of vitamin B12 in the circulation. Our findings supply clinical implications, as they implicate that serum vitamin B12 may play a part in NAFLD risk. Similarly, NAFLD also has a causal impact on elevated serum vitamin B12 concentrations in the circulation.

## Data availability statement

The original contributions presented in the study are included in the article/Supplementary material, further inquiries can be directed to the corresponding authors.

## Author contributions

LF and Y-QH: study concept and design and drafting of the manuscript. LF, Y-QH, and YW: acquisition of data and critical revision of the manuscript for important intellectual content. LF: analysis and interpretation of data. All authors have read and approved the final version of manuscript.

## Funding

This study was supported by grants to LF and Y-QH from the National Natural Science Foundation of China (grants nos. 82204063, 11971117, and 11571082).

## Conflict of interest

The authors declare that the research was conducted in the absence of any commercial or financial relationships that could be construed as a potential conflict of interest.

## Publisher’s note

All claims expressed in this article are solely those of the authors and do not necessarily represent those of their affiliated organizations, or those of the publisher, the editors and the reviewers. Any product that may be evaluated in this article, or claim that may be made by its manufacturer, is not guaranteed or endorsed by the publisher.

## Footnotes

^1^ http://ldsc.broadinstitute.org/
